# Epigenetics and Colorectal Cancer Pathogenesis

**DOI:** 10.3390/cancers5020676

**Published:** 2013-06-05

**Authors:** Kankana Bardhan, Kebin Liu

**Affiliations:** Department of Biochemistry and Molecular Biology, Medical College of Georgia, and Cancer Center, Georgia Regents University, Augusta, GA 30912, USA; E-Mail: kbardhan@gru.edu

**Keywords:** colorectal cancer, DNMT, HDAC, HMTase, epigenetic therapy

## Abstract

Colorectal cancer (CRC) develops through a multistage process that results from the progressive accumulation of genetic mutations, and frequently as a result of mutations in the Wnt signaling pathway. However, it has become evident over the past two decades that epigenetic alterations of the chromatin, particularly the chromatin components in the promoter regions of tumor suppressors and oncogenes, play key roles in CRC pathogenesis. Epigenetic regulation is organized at multiple levels, involving primarily DNA methylation and selective histone modifications in cancer cells. Assessment of the CRC epigenome has revealed that virtually all CRCs have aberrantly methylated genes and that the average CRC methylome has thousands of abnormally methylated genes. Although relatively less is known about the patterns of specific histone modifications in CRC, selective histone modifications and resultant chromatin conformation have been shown to act, in concert with DNA methylation, to regulate gene expression to mediate CRC pathogenesis. Moreover, it is now clear that not only DNA methylation but also histone modifications are reversible processes. The increased understanding of epigenetic regulation of gene expression in the context of CRC pathogenesis has led to development of epigenetic biomarkers for CRC diagnosis and epigenetic drugs for CRC therapy.

## 1. Colorectal Cancer

Cancer is a term used for a disease in which abnormal cells in the human body start dividing and growing without control and are capable of invading other tissues through the blood and lymphatic system, a phenomenon known as metastasis. Colorectal cancer (CRC) is a type of cancer that starts in the colon or rectum portion of large intestine in the gastrointestinal (GI) tract. Recent advances in detection, chemotherapeutic and biological agent-based therapies, combined with liver resection, have dramatically increased the survival rate of CRC patients [1]. However, CRC is still an uncontrollable disease. CRC is the third most commonly diagnosed cancer in both men and women in United States. Overall, the lifetime risk of developing CRC is about 1 in 20 (5.1%), and the mortality rate from CRC is also alarming. In United States, CRC is the third leading cause of cancer-related deaths when men and women are considered separately, and second leading cause when both genders are combined. The American Cancer Society estimates that there will be 142,820 new cases of CRC and CRC will cause 50,830 deaths in the United States in 2013.

CRC develops slowly, over years, even decades. Most CRC starts with the polyps occurring on the epithelial lining of the colon or rectum. These polys may be benign (e.g., hyperplastic polyp), pre-malignant (e.g., tubular adenoma) or malignant (e.g., colorectal adenocarcinoma). It is estimated that about 20% cases of CRC have a family history of CRC. Some genetic syndromes are associated with greater risks of CRC, for example, hereditary nonpolyposis colorectal cancer (HNPCC or Lynch syndrome) accounts for about 3% of people with CRC, and Gardner syndrome and familial adenomatous polyposis (FAP) are nearly always associated with CRC and are the causes of about 1% of all CRC cases. However, the majority of CRC cases are linked to environmental factors rather than heritable genetic changes [2]. Occurrence of CRC may be dependent on various factors, namely diet, life style and other environmental parameters, including environmental and food-borne mutagens [3]. In addition, CRC risk factors also include intestinal microbiota and chronic intestinal inflammation that precede tumor development [4,5,6]. It is estimated that chronic inflammation, likely to be caused by a dysregulated intestinal microbiota, contributes to approximately 20% of all CRC cases [4,7].

## 2. Genetic, Microenvironment and Epigenetic Regulation of CRC Pathogenesis

The mechanisms underlying CRC pathogenesis remain subjects of extensive investigation in the cancer biology field. It is known that CRC results from accumulation of both genetic and epigenetic alterations of the cellular genome that transforms normal glandular epithelium into adenocarcinoma [6]. Furthermore, CRC can further alter the genetic and epigenetic profiles to acquire invasion capability to metastasize to distant organs, primarily to the liver, and to a less degree to the lung via the lymph channels. However, molecular basis of these continuous tumor growth and progression are not fully understood, and various models have been proposed for the CRC pathogenesis. The original Loeb model states that tumor evolution is driven by genetic instability, with the generation of large numbers of random mutations and selections for clones exhibiting a mutator phenotype [8]. Soon afterwards, Nowell’s pioneering work on the role of chromosomal aberrations in tumorigenesis led to the proposal that each cancer’s individual genotype results from multiple rounds of clonal selections. Both of these hypotheses are in lines with Foulds’ multi-step theory of tumorigenesis and the hypothesized colorectal adenoma-carcinoma sequence of events [9]. During the last two decades, Vogelstein’s Darwinian model of adenoma-carcinoma progression has drawn strong support from experimental data [10], his further work on the genetic basis of human HNPCC provided strong evidence for the theorized hypermutable phenotype [11]. These pioneer studies and theories have firmly established the rules of tubular and tubulovillous adenomas as premalignant neoplasms that would progress into invasive adenocarcinoma.

Further advances in the CRC research field have recognized that additional classes of premalignant polyps, such as serrated polyps, also have significant potential for malignant transformation [12]. One of the limitations of the original genetic models of CRC development is that only a fairly limited repertoire of genetic alterations drives the formation of CRC. In addition, in many sporadic colorectal adenocarcinomas, the predicted accumulation of genetic events has been difficult to demonstrate [13]. Also, there is very little evidence for the incremental accretion of genetic events in aging tissues that has been hypothesized to explain the exponential increase in the incidence of CRC with age. It is now clear that there are multiple molecular pathways to CRC, and that these pathways involve both genetic mutations and epigenetic alterations. For example, serrated polyps are associated with microsatellite instability (MSI) and aberrant DNA hypermethylation, whereas tubular adenomas more commonly arise via inactivation of the APC tumor suppressor gene and concurrent genetic alterations resulting from chromosomal instability [14]. 

What further complicates this issue is the recent technology advances that reveal that thousands of molecular alterations exist in the average CRC genome but it is widely believed that only a subset of these alterations drive the cellular and clinical behavior of CRC development. In fact, the molecular heterogeneity of CRC is believed to be one of the factors responsible for the considerable variability in treatment response among patients with the same stage of CRC [15]. Although defined molecular subtypes of CRC do exist, the molecular subgroups cannot be accurately distinguished histologically or clinically at this time. In contrast, the contributions of epigenetic modifications to the pathogenesis of CRC has been highlighted by the identification of a subtype of CRC, the CpG island methylator phenotype (CIMP), that has a distinct epigenome with a high frequency of methylated genes [16]. In addition to DNA hypermethylation that often occurs in the promoter region of tumor suppressor genes, epigenetic regulation of CRC epigenome also includes post-translational histone modifications, primarily histone acetylation and methylation that also play critical roles in regulation of expression of oncogenes and tumor suppressor genes. 

## 3. Genetic Regulation of CRC Development

Like in other types of cancers, the classic view of cancer is that it arises as a consequence of the accumulation of mutations in key tumor suppressor genes or oncogenes, which deregulate the homeostatic functions and cause the transformation of normal cells into cancer cells. Sequencing of colon cancer genomes and an analysis of approximately 13,000 genes identified mutations in the coding sequences of approximately 67 genes in an average colon cancer genome, of which a subset of 12 genes were proposed to be the genes most likely to be involved with cancer formation in individual cancers [17]. The cancer genome project also revealed that most of the cancer genomes harbor hundreds of mutations, but it remains to be determined which of these mutations have a pathogenic role in the formation of cancer (driver mutations) and which are the consequences of this process (passenger mutations). Extensive studies of CRC in the past decades revealed that the majority of genetic alterations are considered harmless passenger mutations, providing no selection advantage. Only approximately 15 alterations are thought to be driver mutations that are functionally important and positively selected for during CRC carcinogenesis [18,19,20]. These driver mutations affect a wide range of cellular functions from proliferation, migration, differentiation, adhesion, cell death, to DNA stability and repair. CRCs can be grouped depending on the types of genomic instability they display. Chromosomal instability (CIN) is characterized by aneuploidy and chromosomal gains or losses, while MSI is identified by the presence of frequent insertion and deletion mutations in the repetitive DNA sequences [21]. Dysplastic aberrant crypt foci can harbor mutations in *APC*, inactivation of which leads to activation of the Wnt pathway, a common mechanism for initiating polyp to cancer progression sequence [22]. Subsequent to the mutation of *APC* or other genes in Wnt pathway, mutations in genes such as *KRAS* or *TP53* occurs and foster the clonal progression of the polyp cells to cancer [22,23]. Progression can also involve mutations in the TGFβ signaling pathway [24]. Mutations in type II TGFβ receptor *(TGFBR2)* gene occur in approximately 30% of CRCs. In addition, mutations affecting other TGFβ signaling pathway members, including *SMAD2*, *SMAD4*, *RUNX3* and *TSP1* have been identified in colon cancers [19,20,24,25,26]. Gene mutations have been proposed to contribute to colon cancer formation through the activation of oncogenes and inactivation of tumor suppressor genes that regulate signaling pathways [27]. For example, *KRAS* is a proto-oncogene that is a downstream effector of EGFR. It signals through BRAF to activate the MAPK pathway. Mutations in *KRAS* or *BRAF* occur in approximately 55–60% of CRCs, aberrantly activating the MAPK signaling pathway, thus inducing proliferation and suppressing apoptosis [28,29]. Less prevalent are germline mutations in DNA mismatch repair genes, *MSH2*, *MSH6*, *MLH1* and *PMS2*, which are associated with frameshift mutations and basepair substitutions in short tandem repeat sequences, causing MSI in the hereditary HNPCC/Lynch syndrome [30]. Beyond point mutations, approximately 85% of CRCs are characterized by CIN with increased chromosomal losses (8p21-pter, 15q11-q21, 17p12-13, 18q12-21) and gains (1q32, 7p, 7q, 8q, 13q, 20p, 20q), and increased loss of heterozygosity [31,32,33]. These observations thus fairly established the roles of genetic alteration, particularly mutations in functional genes regulating proliferation, migration, differentiation, adhesion, cell death, DNA stability and repair, in CRC development. 

## 4. Colon Anatomy and Microenvironment

Consistent with the presence of incontrovertible differences between the ontogeny, morphology, biochemistry and physiology of the different sections of the human colons, compelling data support the concept that proximal and distal colon cancers arise through distinct molecular pathways, thereby establishing the two-colon concept [34]. CIN is implicated in 60–70% colorectal cancers, and more commonly observed in distal compared to proximal colon cancers [35,36]. In contrast, MSI high cancer preferentially occurs in the proximal colon [37]. CpG island methylator phenotype (CIMP) high CRCs with *BRAF* mutations can also be found more frequently in proximal colon [28,29,30,31,32,33,34,35,36,37,38,39,40]. Ogino *et al.* recently reported that in a clinical trial of stage III colon cancer, overall survival was comparatively better in patients with wt *BRAF*/MSI high disease and poorer in those with mutated *BRAF*/microsatellite stable (MSS) disease [41]. 

LaPointe and colleagues undertook an analysis of normal colonic tissue transcripts by DNA microarray [42], and observed that microbial and biochemical components of the luminal ecosystem and interactions occurring within the mucosal-luminal interface are likely to vary gradually along the proximal-distal axis of the colorectum. This underlying continuum was postulated to be responsible for the apparent continuity in the gene expression data. Lifestyle has recently been emerged as another triggering factor of CRC pathogenesis. Campbell *et al.* has described an interesting case control study of body mass index (BMI) and colorectal cancer risk in relation to tumor MSI status [43]. Slattery *et al.* examined 118 MSI high and 696 MSS tumors and found that high BMI was associated with the risk of MSS tumors but not with the risk of MSI high tumors among men, but no differences noted in women population [44]. 

Immune cells are critical components of the tumor microenvironment [45] that act both positively and negatively in tumor development, including CRC pathogenesis [46,47,48,49,50,51]. Extensive studies have been focused on the clinical importance of the host immune response in terms of tumor-infiltrating immune cell activation and function, and demonstrated that the presence and function of specific subsets of lymphocytic cells are consistently associated with a better prognosis in CRC [52,53,54,55,56,57]. In addition, immune response may cause enlargement of lymph nodes, which may contribute to an increase in the recovered lymph node count and thereby more accurate staging of colorectal cancer. In fact, lymphocytic reaction to CRC has been associated with an increased lymph node count [58], and lymph node count has consistently been associated with improved survival of CRC patients [59,60,61,62,63]. However, it was also observed that lymphocytic reactions to tumor were associated with improved prognosis among CRC patients in multivariate models that adjusted for covariates, including MSI, CIMP, and long interspersed nuclear element-1 (LINE-1) hypomethylation, which is independent of lymph node count [64]. In another study with 768 CRC specimens, Nosho *et al.*, observed that CD45RO^+^ T cells density is associated with patient survival (*p* = 0.0032), and MSI-high (*p* < 0.0001) and high-level tumor LINE-1 methylation (*p* = 0.0013) are independently associated with higher CD45RO^+^ T cell density [65]. Furthermore, subsets of tumor-infiltrating T cell density is a prognostic biomarker associated with longer survival On the other hand, CIMP high and MSI tumors are frequently infiltrated by a large number of T cells and are associated with longer patient survival [66,67,68,69,70,71], suggesting the a association between tumor-infiltrating immune cells, tumor cell methylation phenotypes and CRC patient clinical outcomes [52,55], and the potential use of specific epigenetic alterations as molecular targets in CRC immunotherapy [72].

## 5. CRC Epigenetics

The last two decades have witnessed the explosion of information regarding epigenetic alterations in cancer cell genome and cancer development, including CRC development [18,73,74,75]. Extensive studies have been focused on depicting an “epigenetic landscape” since aberrant epigenetic alteration in CRC was first identified in 1980s [76]. The epigenetic landscape determines the chromatin conformation that determines whether the DNA is accessible to transcription factors that control gene expression. An open or relaxed, thus accessible, chromatin conformation facilitates binding of transcription factors to activate or repress transcription initiation of specific genes, while a closed or condensed chromatin state restricts transcription factor binding to the promoter region, thereby preventing transcription factor-dependent transcription regulation and resulting in primarily repression of transcription initiation [77]. Although other epigenetic mechanisms, including nucleosomal occupancy and remodeling, chromatin looping, and noncoding RNAs, also plays important roles in CRC development [18], the major epigenetic mechanisms which are believed to play a crucial role in cancer development includes DNA methylation of cytosine bases in CpG dinucleotides and post-translational modifications of histone proteins that mediate the packaging of DNA into chromatin and thus regulate gene expression through controlling chromatin conformation [77]. Although these multiple epigenetic mechanisms are all involved in CRC pathogenesis and there exist crosstalk between various epigenetic mechanisms, DNA methylation and histone modifications in the gene promoter region are the most extensively studies epigenetic mechanisms, and they also appear to be the primary mediators of CRC epigenetic inheritance in cancer cells [78].

## 6. DNA Hypermethylation

DNA methylation refers to the enzymatic addition of a methyl group to the 5' position of cytosine by DNA methyltransferases (DNMTs) to produce 5-methyl cytosine. The DNMTs work on specific CG dinucleotide sequences, known as CpGs. Over 70% of the cytosine (C) bases in the context of CpG dinucleotides are subjected to methylation. DNA methylation is essential for mammalian development [79] and serves an important function in X-chromosome inactivation [80] and genomic imprinting [81]. The non-cancerous mammalian cell genome contains approximately 70–80% methylated CpGs in the non-promoter region, but CpG islands located around the promoter region of genes are usually unmethylated [82]. Conversely, genome-wide hypomethylation in non-promoter regions and hypermethylation in the promoter region of genes, particularly tumor suppressor and DNA repair genes, are often observed in cancer cells. Hypermethylation contributes to gene silencing and genomic instability and affects apoptosis, DNA repair, and cell-cycle control [82,83].

One of the first recognized epigenetic alterations in CRCs is the depletion of the overall 5-methyl cytosine content (global DNA hypomethylation), which occurs gradually, age-dependently, and early in the process of CRC carcinogenesis [84]. It was speculated and later experimentally demonstrated that hypomethylation provides the incipient cancer cells with a mutator phenotype by destabilizing the karyotype and by promoting loss of heterozygosity (LOH) [85,86,87]. Global DNA hypomethylation occurs predominantly at CpG dinucleotides in repetitive sequences (satellite and LINE repeats, retrotransposons and endogenous retroviral elements), unique sequences including oncogenes and imprinted loci, less commonly in CpG islands and in highly conserved sequences surrounding CpG slands, termed as CpG island shores [88]. In addition, hypomethylation may mimic amplification of oncogenes. Originally, hypomethylation in CRC was hypothesized to be associated with widespread oncogene activation [89]. However, later studies linked DNA hypomethylation to chromosomal instability [90]. Study in mouse models and human CRC revealed that Apc^MIN/+^ mice develop microadenomas associated with LOH of *APC*, probably through increased genetic instability, despite a reduction in the incidence and growth of macroscopic CRCs, whereas tumor hypomethylation is associated with increased chromosomal instability in human CRC [90,91,92,93,94].

**Figure 1:**
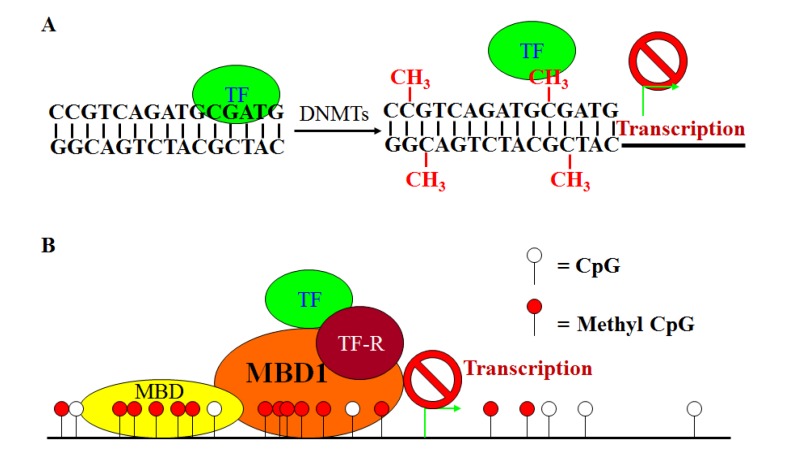
A simplified model of DNA methylation-mediated gene silencing. (**A**). Methylation of the cytosine of the CpG dinucleotides in a gene promoter region within a transcription factor’s binding consensus sequence motif may be recognized by the transcription factor (TF) as a “mutation” and thus results in loss of TF binding and transcription initiation. (**B**). Methyl-CpGs may recruit MBD protein binding to the methylated DNA region in a gene promoter region. The MBD proteins may physically block TF binding to the promoter region or recruit repressors to inhibit TF activity, and thereby silencing gene transcription.

On the other hand, increased expression levels and activity of DNMTs have been reported in human cancers, including cases of CRCs compared with normal tissues [95,96,97,98]. The discovery of allele specific hypermethylation of the retinoblastoma tumor suppressor gene for which Knudson first proposed his “two hit hypothesis” (intragenic mutations and LOH) provide strong evidence for a different mode of gene inactivation, namely promoter hypermethylation [99]. In CRCs, the most extensively characterized epigenetic alteration is gene promoter hypermethylation, which occurs in CpG islands that are often present at the 5' region of approximately 60% of the genes [100]. However, most CpG islands lack methylation in normal colon mucosa, and are independent of the transcriptional status of the gene. Hypermethylation of promoter CpG islands has been observed for numerous tumor suppressor and DNA repair genes and functions equivalently to coding region mutations and deletions [101,102] (Figure 1A). Increased enzymatic DNMT activity is a property of nearly all transformed cells. Three DNMTs have been shown to catalyze DNA methylation in mammalian cells. DNMT3a and DNMT3b transfer methyl groups from S-adenosylmethionine (SAM) to DNA in a *de novo* fashion while DNMT1 does that in a maintenance fashion [103]. *De novo* methylation refers to the methylation of DNA without the use of a DNA template that carries an existing methylation pattern; whereas maintenance methylation occurs during DNA replication, and refers to the replication of the methylation pattern of the unreplicated strand of DNA onto newly replicated DNA strand. 

A prominent mechanism by which DNA methylation regulates gene expression was established in the late 1980s and early 1990s, with the identification of proteins that could bind to mCpGs and recruit protein complexes. A superfamily of proteins, termed methyl CpG-binding domain protein (MBD), is involved in these cooperative interactions by binding with high affinity to mCpGs. The MBD superfamily has three branches: (1) MBD-containing proteins, (2) methyl-CpG binding zinc fingers, and (3) the SRA domain containing proteins [104,105]. The MBD containing proteins is the large group with 11 members (MeCP2, MBD1, MBD2, MBD3, MBD4, MBD5, MBD6, SETDB1, SETDB2, BAZ2A and BAZ2B) [105]. The zinc finger group includes Kaiso, ZBTB4 and ZBTB38, and the SRA domain containing protein group consists of UHRF1 and UHRF2. MBD protein binding to the methylated DNA may physically block transcription factor binding to the promoter region to repress transcription initiation (Figure 1B). MBD binding to the methyl CpG regions may also leads to a cascade of downstream events, including recruiting repressors to the promoter region to inhibit activity of transcription factors [101] and other protein factors that mediate histone modifications and chromatin conformation to remodel the chromatin and thus blocking access of transcription factors to the promoter region of the compacted chromatin structure [18,106] (Figure 1B). The roles of MBD protein in CRC development have been well-documented. Among the known MBD proteins, MeCP2 [107], MBD1 [101,108], MBD2 [108], and MBD4 [109,110,111,112,113,114] have been linked to repression of gene expression in CRC cells.

The Polycomb group (PcG) proteins are another protein complex group that is involved in DNA methylation-mediated gene silencing [115]. Although the multimeric PcG repressive complexes (PRCs), mainly PRC1 and PRC2, can silence gene expression either independently or synergistically, components of the PcG protein complex also interact with DNMTs, and are directly involved in DNMT-dependent DNA hypermethylation *in vivo* [115]. These PcG protein complexes are thought to serve as a recruitment platform for DNMTs [116]. Both PRC1 and PRC2 proteins interact with DNMT1 and DNMT3b, establishing a potential key role for these proteins in catalyzing methylation-associated transcriptional silencing of target genes in cancer cells [116,117,118]. Particularly, PRC-mediated gene transcriptional silencing has been hypothesized to play a role in CRC since many genes that are frequently hypermethylated in CRC are PcG targets [119]. The PRC subunit EZH2 is the most commonly known protein that regulates PcG-mediated transcriptional repression and is often overexpressed in CRC [120].

## 7. Characteristics of DNA Hypermethylation in CRC

DNA methylation-related deficiencies in the mismatch repair (MMR) system can result in mutation rate that is 100-fold greater than normal cells, as direct consequences of an inability to replicate the genome faithfully. One of the best-characterized epigenetic tumor progression pathways in CRC is biallelic promoter CpG island methylation of the mismatch repair gene *MLH1*, associated with MSI-H sporadic CRCs. They are normally stable, but slippage during DNA replication generates insertions/deletions and, if perpetuated, engenders MSI, the hallmark of the replication error phenotype [121]. The defects in MMR first became apparent as germline mutations in human *MutL* and *mutS* homologues *MHL1* and *MSH2*, and more rarely *MSH6*, *PMS1* and *PMS2*, in HNPCC, which bestow an increased rate of progression on adenomatous polyps [11]. It was first described in 2006 that heritable methylation of the promoter of *MSH2* could also cause Lynch syndrome [122]. Ligtenberg *et al.* later found that Alu mediated deletions of the stop codon of the *EPCAM* gene, which is immediately 5' upstream of the start of *MSH2*, result in the somatic methylation and silencing of *MSH2* in those tissues that express *EPCAM* [123]. MSI is also present in 10–15% cases of sporadic CRCs. 

The recognition that a distinct subset of CRCs displays significantly more promoter DNA hypermethylation than others has led to the introduction of CIMP [16,38,124]. CIMP CRCs is associated with older age, female sex, family history of CRC, proximal location in the colon, mucinous cell differentiation, specific precursor lesions (e.g., serrated adenomas), smoking, MSI, *BRAF* and *KRAS* mutations [38,125,126]. CIMP is currently one of the most extensively used features for classifying subgroups of CRC for the purpose of biomarker and therapeutic strategy development [127,128,129,130,131,132]. However, a major challenge in CIMP-based CRC subgroup classification is that there is no general consensus of which specific methylated loci to use to define CIMP subgroups. The most commonly used CIMP markers are the classic panel of *MLH1*, *p16*, *MINT1*, *MINT2*, and *MINT31* [83,127]. In addition to these 5 loci, CIMP marker panel has been extended to include *CACNA1G*, *CRABP1*, *IGF2*, *NEUROG1*, *RUNX3*, *SOCS1*, *HIC1*, *IGFBP3*, or *WRN*, with no consensus on how many markers are required to be positive to define CIMP. Therefore, it is not surprising that clinical study results vary, and even contradict, across studies, depending on which loci marker panel and criteria are used to define the CIMP subgroups [127,128,129,130,131,132,133].

To increase the prognostic value of CIMP markers in CRC, extensive attempts have been made to combine CIMP markers with genetic markers in CRC classification [125,126]. Earlier works have suggested that different epigenetic phenotypes distinguish the mucosa in MSI^+^ and MSI^-^ sporadic colorectal cancers [134]. A defective MMR system (as a part of the CIMP) can lead to the accumulation of point mutations and small deletions and/or insertions in diploid cells [134]. It is suspected that genetic instability may be caused by either a CIMP related MMR mutator phenotype [8] or a phenotype in which hypomethylation dysregulates chromosomal segregation processes in the cancer cells [86,135]. About 50% of adenomas were classified as CIMP^+^, but *MHL1* CGI methylation was not present in these lesions [16]. Second, *MHL1* promoter hypermethylation is also absent from more than half of CIMP^+^ cancer and from 25% of those with MSI. In spite of much conjecturing, *MHL1* DNA hypermethylation in the CIMP is now believed to account for up to 75% of cases of sporadic CRCs with MSI [16]. Several tumor suppressor genes, namely type II TGFβ receptor and *BAX* are downstream targets of MSI in CRCs. Moreover, *MHL1* overexpression has been reported to trigger apoptosis directly [136,137]. Thus, 5' methylation of *MHL1* may influence cell growth, both directly and via its downstream targets. Shen and colleagues have suggested that CIMP CRC can be divided into two distinct classes, CIMP1 and CIMP2, based on analysis of a large panel of methylation marks [125]. A study of 97 primary CRC cases has shown that CIMP1 tumors are often microsatellite instable (80%) and have high *BRAF* mutations (53%), whereas CIMP2 tumors have very high *KRAS* mutations (92%), but rarely have MSI or *BRAF* or *TP53* mutations [20]. Tumors that do not have CIMP have a high frequency of *TP53* mutations (71%) and an intermediate frequency of *KRAS* mutations (33%) [20]. Genome-scale DNA methylation profiling of 125 CRCs identified four DNA methylation subgroups using model-based cluster analyses. These CRC groups were classified as CIMP-high, CIMP-low and non-CIMPs. CIMP-high group tumors exhibit an exceptionally high frequency of cancer-specific DNA hypermethylation and a high rate of mutant *BRAF* (61%), whereas CIMP-low is associated with higher *KRAS* mutation (45%) and has a subset of CIMP-high-associated methylated genes rather than a unique group of CpG islands. The non-CIMP tumors are separated into two distinct clusters based on the association with mutant *TP53* and location of the tumors [138,139]. Overall, although these studies established a certain links between CIMP subgroups and the MSI, *BARF* and *KRAS* genetic phenotypes in CRC patients [125], again, when analyzing clinical outcome results across studies, caution should be taken because the loci marker panel and criteria for CIMP, and the levels of these genetic mutations vary from study to study. 

Inflammation-mediated CRC accounts for approximately 20% cancer. Although it has been postulated that the increased risk of colon cancer in human patients with inflammatory bowel disease (IBD) is due to chronic mucosal inflammation and possibly age-related CIMP, and one study has shown that chronic inflammation is associated with high levels of methylation in human patients with ulcerative colitis (UC) [140], other studies have suggested DNA hypermethylation or increased incidence of CIMP only plays a minor role in IBD-associated CRC as compared to sporadic cancers [141,142,143]. Thus, it is unlikely that IBD-related CRC is via the CIMP pathway. However, it should be noted that although in the individual locus level, MSI is found at an equal prevalence to sporadic cases and loss of expression of *MHL1* is not a frequent event in UC-associated neoplasia, CGI methylation of *MHL1* has been reported in ~15% of IBD-associated CRC [144], suggesting that hypermethylation of individual genes, rather than CIMP, might play a role in inflammation-mediated CRC pathogenesis.

Overall, CRC-specific hypermethylation affects WNT, receptor tyrosine kinase, neurogenic locus notch homolog protein (NOTCH), *TP53*, *PI3K*, retinoic acid and IGF signaling, as well as other signaling pathways that regulate cell cycle, transcription, DNA repair/stability, apoptosis, adhesion, angiogenesis, invasion and metastasis [83,90,91,92,93,94,123]. Therefore, like genetic mutations and alterations, DNA methylation-mediated gene silencing also targets many of the same signaling pathways that are directly involved in CRC development.

## 8. DNA Hypomethylation and CRC Development

As discussed above, DNA hypermethylation is frequently associated with transcriptional silencing of tumor suppressor genes in CRC. Interestingly, global DNA hypomethylation also plays an important role in CRC development, possibly through hypomethylation-induced genomic instability. A classic example of hypomethylation and CRC development is LINE-1. LINE-1 or L1 retrotransposons account for about 17% of human genome and thus their methylation status is an important indicator of global DNA methylation level. However, CpG sites located within the LINE-1 repetitive DNA elements are often heavily methylated in normal cells, which is important for suppression of transposon activity and thus for maintenance of genomic stability [145]. In association with tumorigenesis, CpG sites in these repetitive DNA elements tend to undergo demethylation, leading to generation of diffuse genomic hypomethylation. It has been shown LINE-1 methylation is highly variable among colon cancer and is associated with its poor prognosis [94,146,147]. Although early onset CRC raises the possibility of a hereditary risk factor, it represents no more than 15–20% cases in this group. The remaining 75–80% of early onset CRC is linked to LINE-1 hypomethylation. Recently, a correlation between LINE-1 extreme hypomethylation and earlier age of onset (<60 year old) and poor prognosis was identified [148]. 

The recent advance in technology (e.g., pyrosequencing) allowed detection of subtle differences in average LINE-1 methylation levels among different colon cancer subtypes (e.g., MSI *vs*. MSS). Patient survival data indicates that LINE-1 methylation level as determined by pyrosequencing is associated with patient clinical outcome [147]. Both CIMP high and MSI high phenotypes of colon cancer have been inversely linked to LINE-1 hypomethylation, while in non-MSI high tumors, CIN is correlated with LINE-1 hypomethylation. LINE-1 methylation-low CRC has also been found to be associated with aggressive tumor behavior [147,149,150]. Furthermore, Ogino *et al.* has recently shown that a family history of CRC was associated with a high risk of CRC with low level of LINE-1 methylation [151]. These studies thus firmly established a link between DNA hypomethylation and CRC development in humans.

## 9. Histone Modifications and CRC Development

In addition to DNA methylation-induced transcriptional silencing of genes, post-translational covalent modifications at histone tails constitute another major epigenetic mechanism that regulates chromatin structure and gene expression in human cancers. Chromatin is the state in which DNA is packaged within the cell. The nucleosome is the fundamental unit of chromatin and it is composed of an octamer of the four core histones (H3, H4, H2A, H2B) around which 147 base pairs of DNA are wrapped. The core histones are predominantly globular except for their *N*-terminal “tails”, which are unstructured. A striking feature of histones, and particularly of their *N*-terminal tails, is the various types of modifications of their residues. There are at least seven distinct types of histone modifications, including acetylation, methylation, phosphorylation, ubiquitination, sumoylation, citrullination and ADP-ribosylation [152]. Combinations of these modifications are thought to constitute the so called “histone code” that determine the chromatin conformation and gene expression levels. Among these seven modifications, acetylation and methylation are most extensively characterized ones that are involved in CRC pathogenesis [78,152,153,154]. Simplistically, the function of histone modifications can be divided into two categories: The establishment of global environments and the orchestration of DNA-based biological tasks. To establish a global chromatin environment, modifications help partition the genome into distinct domains such as euchromatin, where DNA is kept “accessible” for transcription, and heterochromatin, where chromatin is “inaccessible” for transcription. To facilitate DNA-based functions, modifications orchestrate the unraveling of chromatin to help the execution of the given function. This may be a very local function, like transcription of a gene or repair of DNA or it may be a genome-wide function, such as DNA replication or chromosome condensation. In mammals, silent heterochromatin state is associated with low levels of histone acetylation and the actively transcribed euchromatin often has high levels of histone acetylation [103,152,155,156]. 

Histone acetylation is reversible modifications of lysine residues on histone “tails” and is controlled by histone acetyltransferases (HATs) and histone deacetylases (HDACs) that typically act as transcriptional co-activators or co-repressors, respectively. Of all the known modifications, acetylation has the most potential to unfold chromatin since it neutralizes the basic charge of the lysine. HATs are divided into three main families: GNAT, MYST and CBP/p300 [157]. Most of the acetylation sites characterized till date fall within the *N*-terminal tail of histones, which are more accessible for modification. However, a lysine within the core domain of H3 (K56) has recently been found to be acetylated. The K56 residue is facing towards the major groove of the DNA within the nucleosome, so it is in a particularly good position to affect histone/DNA interactions when acetylated. A yeast protein SPT10 may mediate acetylation of H3K56 at the promoters of histone genes to regulate gene expression [158], whereas Rtt109 mediates this modification more globally [159,160,161]. The reversal of acetylation correlates with transcriptional repression. There are three distinct families of HDACs: The class I and class II HDACs and the class III NAD-dependent enzymes of the Sir family [162]. They are involved in multiple signaling pathways and they are present in numerous repressive chromatin complexes. In general, these enzymes do not appear to show much specificity for a particular acetyl group.

HDACs are the most extensively characterized proteins among the histone modification enzymes that play key roles in CRC development. Multiple class I HDACs are up-regulated in a subset of CRCs, including HDAC1 in 36.4%, HDAC2 in 57.9%, and HDAC3 in 72.9% of CRC specimens, and high HDAC expression has been shown to be associated with reduced patient survival in CRC [163]. Nuclear expression of HDAC2 was observed in 81.9% of colorectal carcinoma, 62.1% colorectal adenoma and 53.1% of normal tissues, respectively [164]. The overexpression of HDAC2 is accompanied the hypoacetylation at H4K12 and H3K18 histones during adenoma to carcinoma progression. In addition, over-expression of HAT-related protein CREB binding protein (CBP) in CRC tissues is correlated with long-term survival, while up-regulation of p300 is correlated with poor prognosis [165]. The class III HDAC, SIRT1, is shown to be overexpressed in 40% of CRCs associated with CIMP-high and MSI-H tumors in a study of 485 CRC cases. Inhibition of SIRT1 can reactivate the transcriptionally silenced genes, suggesting a possible therapeutic approach to reverse epigenetic alteration induced silencing process of many genes [166].

Methylation is another major post-translational modification of histones that occurs both lysine and arginine residues of the *N*-terminal tail of histones. Like histone acetylation, histone methylation is also now appreciated as a reversible process [152,155,156]. Its homeostasis is mediated by two antagonizing groups of enzymes, histone methyltransferases (HMTases) and histone demethylases (HDMs), which install and remove histone methylation marks, respectively, in a site-specific manner [167,168]. To date, more than twenty lysine and arginine HMTases have been identified. Each of the HMTases, either acts alone or in complex with other HMTases, catalyzes site-specific histone methylation. For example, Histone H3 lysine 4 (H3K4) methylation is established by SET1 and MLL [168], and removed by the lysine specific histone demethylase 1 (LSD1) and jumonji AT-rich interactive domain 1 (*JARID1*) family of HDMs [169]. For histone H3, methylation has been observed at multiple lysine sites, including H3K4, K9, K27, K36 and K79, and addition of up to three methyl groups at each lysine produces a total of four methyl states: Unmethylated, monomethylated, dimethylated and trimethylated. These histone methylation states exhibit a distinct distribution pattern in the mammalian genome [170]. H3K4 trimethylation (H3K4me3) is strongly associated with transcriptional competence and activation, with the highest levels observed near transcriptional start sites of highly expressed genes. Two other methylation sites on histones, H3K36 and H3K79, are implicated in activation of transcription [171,172]. On the contrary, H3K27 trimethylation (H3K27me3) is frequently associated with gene silencing, especially the repression of unwanted differentiation programs during lineage specification [170,173,174]. Two other lysine methylation sites that are connected to transcriptional repression are H3K9 and H4K20 [175]. 

The establishment of an appropriate pattern of histone methylation is not only crucial for normal development and differentiation, but is also intimately associated with tumor initiation and development. For example, DOT1l is an activator of the Wnt-dependent transcription in CRC cells [176]. The PRC protein EZH2 also functions as HMTase that is frequently found to be overexpressed in various solid tumors, including colon cancer [177,178]. The oncogenic function of EZH2 has been mechanistically attributed to the silencing of the tumor suppressor genes, including *INK4B-ARF-INK4A*, E-cadherin, *p57 ^KIP2^*, *p27* [179], *BRCA1* [180] and adrenergic receptor β2 [181]. Elevated *MLL2* expression level has been observed in poorly differentiated and more invasive colon carcinoma cell lines, and in colon carcinoma tissues as compared to the corresponding adjacent normal colonic epithelium [182]. MLL2 is primarily regarded as a nuclear protein with nuclear localization signal. Colonic cell lines derived from highly invasive tumors, exhibited altered sub-cellular distribution and proteolytic processing of MLL2 compared to the non-tumor/less invasive tumor cell lines. Thus, this deregulated expression, altered distribution as well as aberrant proteolytic processing of MLL2 may be linked to tumorigenesis in colonic tissues by adversely impacting MLL2 mediated histone methylation activities, and in turn, disrupt downstream gene signaling, which potentially involves cell cycle or cell proliferation activities [182]. SUV39H1 is a HMTase that specifically methylates H3K9 at the pericentric heterochromatin. Elevated SUV39H1 expression level was found in 54 of 219 CRC patients specimens examined. More interestingly, a significant correlation between SUV39H1 mRNA level and DNMT1 mRNA level was also observed in those CRC patient specimens, suggesting a potential coordination of H3K9 methylation and DNA methylation in CRC cells under pathophysiological conditions [183]. Overall, these studies demonstrated that altered expression and functions of HMTase are characteristics of cancer, including CRC. 

## 10. microRNAs as Epigenetic Regulators in CRC

Non-coding RNAs, particularly microRNAs (miRNAs), are mechanistically involved in controlling the expression of various cancer-associated genes, and their expression may be altered in cancer, including CRC. miRNAs are single stranded, evolutionary conserved, small RNA molecules (19–25 ribonucleotides) that mediate post-transcriptional gene repression. Typically primary miRNA genes are transcribed by RNA polymerase II in the nucleus, processed to precursor miRNA by Drosha, and transported to the cytoplasm, whether they undergo further processing by the ribonuclease III enzyme Dicer, resulting in mature miRNAs that are incorporated into the RNA induced silencing complex. 

miRNAs act as endogenous suppressor of gene expression through imperfect binding of the RNA induced silencing complex to the 3' untranslated regions of target mRNAs and induce either mRNA degradation or translational repression. So miRNAs might either be oncogenic (*onco*miRs), inhibiting expression of target tumor suppressor genes or tumor promoting (*ts*miRs), inhibiting the expression of the oncogenes [184]. Soon after the recognition of the involvement of miRNAs in human leukemia, two miRNAs, miR-143 and miR-145, were reported to be significantly down regulated in colorectal adenomas and carcinomas in comparison to the normal colon tissue [17]. Subsequent functional studies have revealed that these two miRNAs act as tumor suppressor in the colon [185,186,187]. More recently, a genomic-wide analysis identified 64 miRNAs to be robustly methylated in human colon carcinoma cells [188], suggesting that miRNA expression is also regulated by epigenetic mechanism such as DNA methylation in human CRC.

Serial analysis of miRNA gene expression (miRAGE) led to the development of microRNAome signatures that identified 133 novel miRNAs in CRC [17]. The major signaling pathways and cellular processes that are regulated by miRNAs in colon neoplasia include β-catenin/WNT signaling (miR-135a/b, miR-139, miR-145, miR17-92) [189,190,191,192,193,194], proliferation (let-7 family, miR-18a, miR-21, miR-126, miR-143, miR-200c) [189,192,195], apoptosis (miR-34a, miR-133b, miR-195) [192,195], cell cycle control (miR34a, miR-192, miR-215, miR-675) [192,195], p53 signaling (miR-34b/c) [196], differentiation (miR-141, miR-200c) [197,198], and migration and invasion (miR-126, miR-143, miR-196a, miR-200a/b/c, miR-373, miR-520c) [192,195,197]. 

## 11. The Crosstalk Network among DNMT, HAT, HDAC, HMTase and HDM: A Complex Network

**Figure 2:**
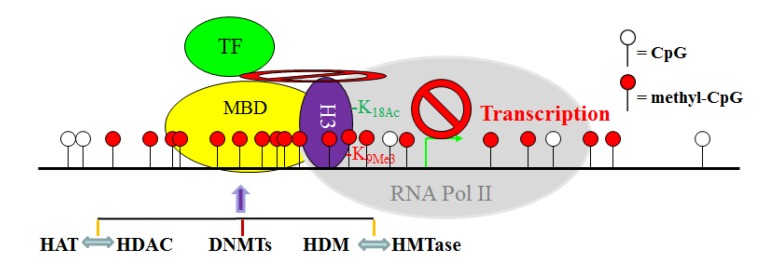
The crosstalk network between the DNA methylation and histone modification systems in the context of gene transcription initiation in CRC cells. DNMTs directly catalyze CpG methylation, whereas HAT and HDAC, as well as HDM and HMTase, antagonize each other to determine specific acetylation and methylation of specific histone residues. However, DNMT, HAT, HDAC, HMTase, and HDM may directly interact with each other while associated with chromation in a gene promoter region. These specific epigenetic modifications of DNA and histone, in concert with specific protein-protein physical interactions between these epigenetic modification enzymes, define a transcriptionally repressive chromatin conformation to block/inhibit transcription factor (TF)-dependent transcription initiation by RNA polymerase (RNA Pol II).

Although DNA methylation, histone acetylation and methylation are catalyzed by distinct enzymes, these epigenetic modifications are apparently tightly coordinated. It is known that DNMTs, MBDs, HATs, HDACs, HMTases and HDMs often form protein complexes in the promoter regions of certain genes in CRC cells [103,183,199] (Figure 2). One such example is the epigenetic regulation of expression of the *MLH1* gene in CRC cells [200]. Deacetylation of H3K9 and simultaneous di-methylation of H3K9 in the *MLH1* promoter mediate the silencing of this gene, but treatment with DNMT inhibitor 5'-aza-cytidine (decitabine) lead to the complete reversal of *MLH1* DNA methylation and the corresponding histone code [200]. Moreover, it has been shown that hypoacetylation of histone together with histone methylation is the first event in gene silencing process, followed by hypermethylation of CpG sites, suggesting that CpG island DNA methylation is a late event that may serve to irrevocably lock the silenced genes [103]. Therefore, DNA methylation, histone acetylation and methylation appear to form a mutually reinforcing silencing loop that contributes to tumor-suppressor gene inactivation in CRCs. Thus, elucidating the molecular mechanisms underlying DNA methylation and histone modifications as a driving force in CRC pathogenesis opens new fields of research to identify molecular targets for CRC therapy. 

## 12. Epigenetic Therapy

It is now a well-established concept that epigenetic alterations can be the driver events in the pathogenesis of CRC, and these epigenetic events cooperate with genetic alterations to promote the progression of normal colonic epithelial cells to cancerous cells and metastasis [18,201,202,203,204]. However, unlike genetic mutations that are essentially fixed forever, epigenetic modifications, including DNA methylation and histone acetylation and methylation, are intrinsically reversible by the cell and extrinsically reversible by pharmacological means. This makes epigenetic marks attractive molecular targets for therapeutic interventions in cancer therapy [205]. 

DNMT modulators represent a class of promising agents in epigenetic-based cancer therapies, and several epigenetic agents interfering with DNMT activity are currently in pre-clinical and clinical trials [205,206,207,208,209,210,211,212,213]. Nucleoside analogs are the most important class of DNMT inhibitors. Once incorporated into DNA, they form covalent bonds with the DNMTs to trap the enzymes to make them unavailable for further methylation, thus resulting in demethylation of replicating nascent DNA [214]. Two cytidine analogs, decitabine and azacytidine, inhibit DNMT activity upon incorporation into DNA, resulting in the loss of DNA methylation. Both of these agents are approved by FDA for use in patients with myelodysplastic syndrome, and are currently being tested extensively in human patients with solid cancers, including CRC. These DNMT inhibitors were initially used at or near their maximally tolerated doses (MTD) in human patients with solid cancers and were observed to be associated with extensive toxicity and minimal efficacy [215,216]. However, later studies indicated that both decitabine and azacytidine at a dose that is much lower than their MTD are effective in inhibiting tumor-specific DNA hypermethylation [216]. Blocking the enzymatic activity of DNMTs by using small molecule inhibitors is another strategy to achieve DNA demethylation [217]. Furthermore, natural products, such as dietary catechol-containing polyphenols, are currently receiving much attention [218,219,220,221]. However, a major concern associated with its use is product standardization and hence creates discrepancies in their reported demethylating activity.

HDAC inhibitors are the most extensively studied and explored agents in the epigenetic drug family. There are at least 20 structurally different HDAC inhibitors that are currently in clinical trials as monotherapy or combinational therapy for treatment of human cancers, including CRC, and two have been approved in the treatment of cutaneous T-cell lymphoma [222]. Hydroxamic acids include the majority of HDAC inhibitors that are currently in clinical trials. Vorinostat is a FDA-approved hydroamic acid HDAC inhibitor that acts through facilitating the hydroxamic moiety to chelate zinc ion located in the catalytic pocket of HDACs, thereby inhibiting deacetylation to result in accumulation of hyperacetylated histones. It has been shown that one of the mechanisms underlying vorinostat action is to modulate Fas expression and other apoptosis-related genes to potentially sensitize CRC cells to FasL-induced apoptosis by tumor-specific T lymphocytes *in vivo* [223,224]. 

In contrast to DNMT and HDAC inhibitors, the search for HMTase inhibitors is still in its infancy [225,226]. The first group of HMTase inhibitors is S-adenosylmethionine (SAM) and its analog S-adenosyl homocysteine (SAH). However, these inhibitors are problematic since they are non-specific and inhibit not only HMTases but also other enzymes which use SAM as a co-factor. Chaetocin, BIX-01294, UNC0638, BRD4770, EPZ004777, AZ505, and PDB4e47 are other relatively selective HMTase inhibitors identified by random chemical library screening [206,207,208,209,210,211]. Overall, although it is much understudied and explored as compared to DNMT and HDAC inhibitors, the development of HMTase and HDM inhibitors is an important task that will likely to take off in the years to come.

**Figure 3:**
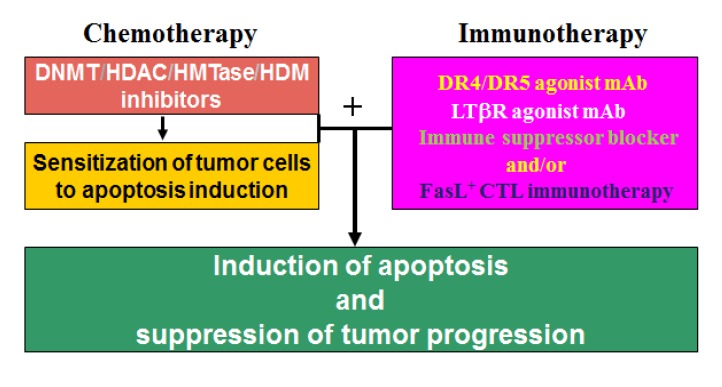
A “one-two punch” strategy of combined chemotherapy and immunotherapy to suppress metastatic human CRC. The apoptotic-resistant CRC cells are first targeted with apoptosis-sensitizing epigenetic drug(s) (e.g., decitabine and vorinostat) to sensitize the tumor cells to apoptosis. Once “sensitized”, patients are then treated with immune effector molecules or FasL^+^ tumor-specific CTLs that promote apoptosis to effectively destruct the tumors.

As discussed above, DNA methylation and histone modifications coordinate to mediate gene expression in cancer cells. Therefore, it is not surprising that single-agent activities of either DNMT inhibitors or HDAC inhibitors in patients with solid tumors are modest [215,227]. Although HDAC inhibition alone induces the expression of many genes, the re-expression of DNA hypermethylation-silenced genes is not typically observed without DNA methylation inhibition in solid cancer cells. In contrast, combinational epigenetic therapy targeting both DNMT and HDAC activity synergistically re-activates gene expression and result in effective tumor suppression [216]. A good example of the combined DNMT and HDAC therapy is a recent study of the death receptor Fas [228]. Fas is a death receptor on mammalian cells, including tumor cells, and acquisition of resistance to Fas-mediated apoptosis is a hallmark of human cancer in general [229]. Fas is essential for FasL-exerted cytotoxicity of the tumor-specific cytotoxic T lymphocytes (CTL) during cancer immune surveillance [230], and thus plays a critical role in suppression of spontaneous tumor development by the host immune system [230,231]. However, CRC cells often silence Fas expression and/or acquire an apoptosis-resistant phenotype to evade Fas-mediated apoptosis [45,229]. For example, Fas is constitutively expressed at high levels in normal human colon tissues, but in human primary colorectal carcinoma, Fas expression is often diminished, and complete loss of Fas expression is often observed in metastatic human colorectal carcinoma [232]. Thus, resistance to Fas-mediated apoptosis is a major obstacle of Fas-based CTL immunotherapy against metastatic human CRC. Apparently, down-regulation of Fas is mediated by epigenetic mechanisms in human colon carcinoma cells since combinational therapy with DNMT and HDAC inhibitors synergistically activate Fas transcription, as well as pro-apoptotic genes such as BNIP3 and Bik, thereby resulting in increased CRC cell sensitivity to apoptosis induction *in vitro* and growth inhibition *in vivo* [228]. These observations thus demonstrated the potential of combined chemotherapy and Fas/FasL-based CTL immunotherapy as an effective approach in suppression of human CRC progression. In general, induction of apoptosis is a major effector mechanism that the tumor-specific CTL use to eliminate tumor [233,234,235]. However, cancer cells often acquire an apoptotic resistant phenotype through various mechanisms, including epigenetic mechanisms, to evade the anti-tumor immunity [102,229,236]. Thus, a “one-two punch” approach may be an effective strategy in CRC therapy: First, sublethal and subtoxic doses of chemotherapeutic agents, such as epigenetic inhibitors decitabine and vorinostat, can be used as an effective and yet less toxic sensitizer to sensitize the apoptosis-resistant CRC cells. Once sensitized, the tumor cells can then be eliminated effectively by immune effector molecules (e.g., TRAIL, FasL, or LTβR agonist mAb [47,237]) or by TCTL immunotherapy (Figure 3). 

Overall, epigenetic agents, primarily DNMT and HDAC inhibitors, have undergone major preclinical investigations and extensive clinical trials, either alone or in combinations with each other or conventional chemotherapeutic agents, for their efficacy to treat various types of solid cancers, including CRC [118,238]. However, the current challenge is to find selective epigenetic agents that demethylate or acetylate specific target(s) to reduce toxicity and improve the response to therapies.

## 13. Epigenetic Regulation of Chemosistance in CRC

There are growing evidences to support the notion that epigenetic alterations play a major role in drug resistance in different solid tumors, including CRCs [239]. Therefore, epigenetic agents may also be used as sensitization agents to overcome CRC drug resistance to increase the efficacy of conventional chemotherapeutic agent in the clinic. 5-Fluorouracil (5-FU) is the standard therapy for human patients with colorectal cancer. Although it was developed more than 50 years ago, 5-FU is still the backbone of treatment for patients with colorectal cancer, especially metastatic colorectal cancer [205,240]. However, CRC resistance to 5-FU, including the more recently developed oral fluoropyrimidines, is often inevitable in human CRC patients [205,240], and therefore elucidating the underlying molecular mechanisms of CRC 5-FU resistance is of great significance for the development of molecular target-based strategies to overcome drug resistance, currently the single most significant challenge in human CRC management [240].

5-FU is an analogue of uracil with a fluorine atom at the C-5 position in place of hydrogen, and enters the mammalian cells rapidly using the same facilitated transport mechanism as uracil [241]. Both normal and tumor cells metabolize 5-FU to 5-fluoro-2'-deoxyuridine monophosphate (FdUMP), 5-fluoro-2'-deoxyuridine triphosphate (FdUTP) and 5-fluorouridine triphosphate (FUTP). Although these 5-FU metabolites may incorporate into DNA and RNA in human CRC cells to cause apoptosis [242,243], 5-FU likely exerts its anti-cancer activity primarily through inhibition of thymidylate synthase (TS), the rate-limiting enzyme in the pyrimidine nucleotide synthesis [205,240,244]. TS catalyzes the methylation of deoxyuridine monophosphate (dUMP), with 5,10-methylenetetrahydrofolate (CH2THF) as the methyl donor, to form deoxythymidine monophosphate (dTMP). This reaction provides the sole *de novo* source of thymidylate. Thymidylate is the necessary precursor of thymidine triphosphate (dTTP) that is an essential precursor for DNA replication and repair [245,246]. The 5-FU metabolite FdUMP, together with CH2THF as a co-factor, binds to TS to form a covalently bound ternary complex, thereby blocking of the normal substrate dUMP binding to inhibit dTMP synthesis [247]. Depletion of dTMP leads consequent depletion of dTTP, resulting in perturbations in the levels of dATP, dGTP and dCTP through various feedback mechanisms to disrupt DNA replication and repair. Disruption of DNA synthesis and repair in the rapidly replicating tumor cells results in severe DNA damage and subsequently cellular apoptosis [240,248,249]. Therefore, 5-FU is considered as a S phase-specific anti-cancer cytotoxic agent.

Considering the critical role of TS in CRC 5-FU resistance, it is not surprising that increased TS expression is widely accepted as a major molecular mechanism for 5-FU resistance in cancer cells, particularly in CRC cells. Compelling experimental data have shown that increased TS levels in CRC cells is mediated by multiple genetic mechanisms, including amplification of *TYMS*, the gene coding for TS [250,251,252,253], and transcriptional regulation of *TYMS* by transcriptional factors whose expression level is altered in tumor cells [254,255,256]. However, although increased TS level has been well-demonstrated as a genetically regulated mechanism that underlies 5-FU resistance in CRC cells, recent studies have started to shed light into the role of epigenetic events in regulation of CRC 5-FU resistance [257,258]. DNA methylation has been implied in CRC 5-FU resistance primarily through two ways: First, DNA methylation mediates the expression of genes involved in 5-FU metabolism and action [201,243,259]; and second, DNA methylation also regulates the expression of key apoptosis-regulating genes that are essential for 5-FU-induced apoptosis in CRC cells. Uridine monophosphate (UMP)/cytidine monophosphate (CMP) kinase (UMPK) not only catalyzes an important step in the phosphorylation of UTP, CTP and dCTP, but is also involved in the necessary phosphorylation by cellular kinases of nucleoside analogs used in antiviral therapies, including activation of 5-FU to 5-FUTP [260]. It was observed that UMPK expression level is lower in 5-FU-resistant human colon carcinoma cells, and decitabine treatment increases UMPK expression level both *in vitro* and *in vivo* and colon carcinoma cell sensitivity to 5-FU [243,259], suggesting that silencing UMPK expression by DNA methylation might be an underlying molecular mechanism of acquired CRC resistance to 5-FU during treatment. However, although the methylation level the CpGs in the UMPK promoter region is enriched in the resistant CRC cells, it remains to be determined whether DNA methylation plays a direct role in UMPK down-regulation in 5-FU-resistant CRC cells due to the low level of CpGs in the UMPK promoter region [205]. Secreted protein acidic and rich in cysteine (osteonectin, SPARC) is a tumor suppressor gene that has been shown to mediate CRC cell sensitivity to 5-FU and is down-regulated in 5-FU-resistant CRC cells [203,204]. Interestingly, SPARC promoter is hypermethylated and SPARC expression is silenced by DNA methylation in human CRC cells [202,261], and decitabine can effectively up-regulates SPARC expression and overcome CRC cell resistance to 5-FU [261]. However, the molecular mechanism underlying SPARC function in 5-FU resistance in CRC cells requires further investigation.

5-FU suppresses CRC through induction of apoptosis, particularly through activation of the intrinsic apoptosis pathway. Thus apoptosis mediators are critically important for 5-FU sensitivity. Several apoptosis regulatory genes, including *Bik, BNIP3* and *DAPK*, have been shown to be silenced by their promoter DNA methylation in CRC cells [262,263,264]. Furthermore, it was observed that the response rate to 5-FU therapy is significantly lower in CRC patients with methylation of either *DAPK* or *BNIP3*, or both, than in those without methylation. Both progression-free survival and overall survival time are significantly shorter in patients with methylation of either or both of these genes than in those without [264]. Therefore, it is apparent that CRC cells use DNA methylation to silence *BNIP3* and *DAPK* to acquire an apoptosis-resistant phenotype to resist 5-FU, and thereby specific targeting of these DNA methylation-silenced genes might potentially be an effective and yet less toxic approach to overcome CRC resistance to 5-FU. 

Alteration of histone acetylation level and patterns is another layer of epigenetic mechanism underlying 5-FU resistance in CRC cells [205,265]. The most noticeable example is regulation of *TYMS* expression by HDAC inhibitors and sensitization of CRC cells to 5-FU [265,266] In addition to its genetic regulation, it is apparent that *TYMS* expression is also regulated by histone acetylation. Various HDAC inhibitors, including the FDA-approved vorinostat, have been shown to effectively down-regulate *TYMS* expression and sensitized CRC cells to 5-FU-induced apoptosis [267,268,269]. These observations thus established the concept that HDAC inhibitors are potentially effective adjunct agents in human cancer therapy to overcome 5-FU resistance.

Although histone methylation plays an important role in human disease, including CRC pathogenesis, and HMTase and demethylase inhibitors are considered emerging therapeutic agents [225]. The role of histone methylation in CRC resistance to 5-FU therapy is unclear. In addition, it is known that DNA methylation and histone acetylation often act in concert to mediate gene expression in CRC cells [228]. Therefore, much more efforts are needed to understand the various epigenetic mechanisms, especially the crosstalk between various epigenetic mechanisms in the context of 5-FU resistance in CRC cells, which is one of the frontlines in CRC research and drug development.

## 14. Conclusions

CRC development is an multiple step event that is mediated not only by genetic mutations, but also by microenvironment factors and epigenetic alterations. While genetic and microenvironment factors may play a dominant role in CRC tumorigensis, recent studies demonstrated that epigenetic alterations are also drivers of CRC initiation and progression. More importantly, unlike genetic alterations which are fixed forever, epigenetic alterations are reversible processes and thus can be targeted by specific therapeutic agents. Despite some discouraging early trial results of DNMT and HDAC inhibitors in human patients with solid tumors, DNMT and HDAC inhibitors are currently extensively tested in human CRC patients in clinical trials, and it seems that sublethal and subtoxic doses of these epigenetic agents may be more effective if used as sensitizers, rather as direct inducers of cell death. Therefore, optimization of dosage and timing, together with development of target-specific new generation of epigenetic inhibitors that can effectively sensitize CRC cells to host immune cells or to standard chemotherapeutic agents appear to be the direction of epigenetic agent-based cancer therapy. In addition, although development and therapeutic use of HMTase and HDM inhibitors are still in its infancy, selective inhibitors of specific HMTases and HDMs are promising next generation “epi-drugs” that are waiting to be discovered and developed in the years to come.
